# Surface Morphology and Microstructure Evolution of Single Crystal Diamond during Different Homoepitaxial Growth Stages

**DOI:** 10.3390/ma14205964

**Published:** 2021-10-11

**Authors:** Guoqing Shao, Juan Wang, Shumiao Zhang, Yanfeng Wang, Wei Wang, Hong-Xing Wang

**Affiliations:** Key Laboratory of Physical Electronics and Devices, Ministry of Education, School of Electronic Science and Engineering, Xi’an Jiaotong University, Xi’an 710049, China; shao_gq@foxmail.com (G.S.); wj17032021@stu.xjtu.edu.cn (J.W.); ZhangShumiao@stu.xjtu.edu.cn (S.Z.); yanfengwang@stu.xjtu.edu.cn (Y.W.); wei_wang2014@mail.xjtu.edu.cn (W.W.)

**Keywords:** hillock growth, step-flow growth, surface morphology, microstructure, crystal quality

## Abstract

Homoepitaxial growth of step-flow single crystal diamond was performed by microwave plasma chemical vapor deposition system on high-pressure high-temperature diamond substrate. A coarse surface morphology with isolated particles was firstly deposited on diamond substrate as an interlayer under hillock growth model. Then, the growth model was changed to step-flow growth model for growing step-flow single crystal diamond layer on this hillock interlayer. Furthermore, the surface morphology evolution, cross-section and surface microstructure, and crystal quality of grown diamond were evaluated by scanning electron microscopy, high-resolution transmission electron microcopy, and Raman and photoluminescence spectroscopy. It was found that the surface morphology varied with deposition time under step-flow growth parameters. The cross-section topography exhibited obvious inhomogeneity in crystal structure. Additionally, the diamond growth mechanism from the microscopic point of view was revealed to illustrate the morphological and structural evolution.

## 1. Introduction

Due to its excellent electrical properties, such as wide band-gap, high carrier mobility, high saturation velocity, low dielectric constant, and high breakdown field, diamond is considered as a potential candidate material for fabrication of durable diamond-based devices with high performances of high-frequency and high-power, which can operate in harsh environments [1,2,3,4,5]. Furthermore, diamond possesses the highest thermal conductivity among materials, higher than GaN and SiC, and is the most ideal heat spreading material for power devices. However, the high and stable performance of diamond-based devices is directly linked to large and high-quality diamond films [6]. For several decades, researchers have used natural and high-pressure, high-temperature (HPHT) diamonds to develop electronic devices. Natural and HPHT diamonds are limited by their high cost, low growth rate, and small size [7].

In 2002, Yan et al. [8] introduced a small amount of N_2_ into microwave plasma chemical vapor deposition chamber and increased growth rate to 150 μm/h, which presented an interesting prospect for the development of large-area single crystal diamond (SCD). In this way, the Japanese group developed a mosaic SCD substrate with a size of 40 × 60 mm^2^ by connecting several “clone wafers” [9], and they have already put 20 × 20 mm^2^ SCD wafers into production. Recently, a German Scientist has developed a freestanding SCD substrate with a diameter of 92 mm by heteroepitaxy on Ir/YSZ/Si(001) [10]. However, the dislocations [11,12,13,14] and stress [15,16] in diamond films are still a major issue in developing large and high-quality diamonds. In order to improve these phenomena, several techniques have been used [17,18,19,20,21,22,23,24]. The remarkable functional properties of diamonds depend not only on their physical and chemical properties but also on their surface morphology. However, in the epitaxial growth of diamond films, the growth mechanisms determine the surface morphologies and eventually the properties of diamond films. Several papers reported the surface morphology of homoepitaxial diamond films varying with deposition parameters [25,26,27,28]. However, there are few reports on homoepitaxial diamond surface morphology evolution from microstructure by changing the growth models. Thus, further studies are necessary for better understanding the internal transformation of the homoepitaxial diamond growth mechanism by varying the growth models. So far, a great deal of research has been carried out on growth models of hillock growth or the step-flow growth mechanism by varying growth parameters, but as far as we are aware, research on the transformation of homoepitaxial diamond growth by varying the growth models from hillock growth to step-flow growth have not ever been conducted sufficiently. Most importantly, this study is expected to make a significant contribution not only towards establishing a growth model for high-quality homoepitaxial diamond films from the point of view of surface morphologies but also towards a thorough understanding of growth mechanism of epitaxial diamond films from hillock to step-flow.

In this work, the diamond hillock interlayer with a granular surface morphology was introduced on a HPHT substrate to grow a step-flow SCD layer by varying the growth parameters in a microwave plasma chemical vapor deposition reactor. We explored the growth mechanism and detailed evolution of the surface morphology, microstructures, and crystal quality of grown diamonds by changing the growth models from hillock growth to step-flow growth. The transformation of homoepitaxial diamond growth by varying the growth models from hillock growth to step-flow growth have been of little concern for understanding the growth mechanisms of diamond films. This work provides a thorough understanding of the internal transformation of homoepitaxial diamond growth by varying the growth models.

## 2. Materials and Methods

Homoepitaxial growth of diamonds was carried out using a 2.45 GHz, 5 kW microwave plasma chemical vapor deposition reactor under our optimized growth conditions. In this experiment, a mechanically polished HPHT Ib-type (001)-oriented 3 × 3 × 0.5 mm^3^ diamond was used as substrate.

Prior to growth, the substrate was pre-treated by H_2_/O_2_ (500:5) plasma at 950 °C for 30 min to eliminate the surface impurities and lattice defects caused by mechanical polishing procedure [29]. Then, the hillock interlayer was deposited on substrate by using microwave plasma chemical vapor deposition apparatus under the hillock growth parameters. The hillock growth parameters were as follows: H_2_ flow rate, CH_4_/H_2_ ratio, and O_2_/H_2_ ratio; chamber pressure and surface temperature were 500 sccm, 10%, 1%, 160 Torr, and 1160–1170 °C, respectively. Afterwards, the growth conditions were changed from hillock growth parameters to step-flow growth parameters. The step-flow growth parameters were as follows: the gas flow rate was 800 sccm for H_2_, CH_4_ concentration was 9%, O_2_ concentration was 1%, the reaction pressure was 120 Torr, and the substrate temperature was 1115–1125 °C. In addition, a small amount of N_2_ was introduced into the feed gas mixture during diamond growth to maintain good morphology in (001) growth sectors [30,31].

To investigate the growth mechanism, the sample was set in microwave plasma chemical vapor deposition reactor for growth in multi runs. During the experimental process, the substrate temperature was measured by a two-color infrared emission thermometer through a quartz window of microwave plasma chemical vapor deposition reactor. The growth temperature was controlled by the microwave power. Surface morphology and cross-section structure of grown diamond were evaluated by field emission scanning electron microscopy (FE-SEM, QUANTA FEG 250, FEI Ltd., Hillsboro, USA) and high-resolution transmission electron microcopy (HRTEM, JEM-2200FS, JEOL Ltd., Tokyo, Japan). The samples for TEM observation were prepared by focused ion beam technique. The Raman and photoluminescence (PL) spectroscopy measurement were performed at room temperature using a LabRAM HR Evolution spectrometer (Nd:YAG laser, Horiba Scientific, Paris, France, operating at 532 nm) in a confocal configuration with spectral resolution of 0.35 cm^−1^.

## 3. Results and Discussion

**Figure 1:**
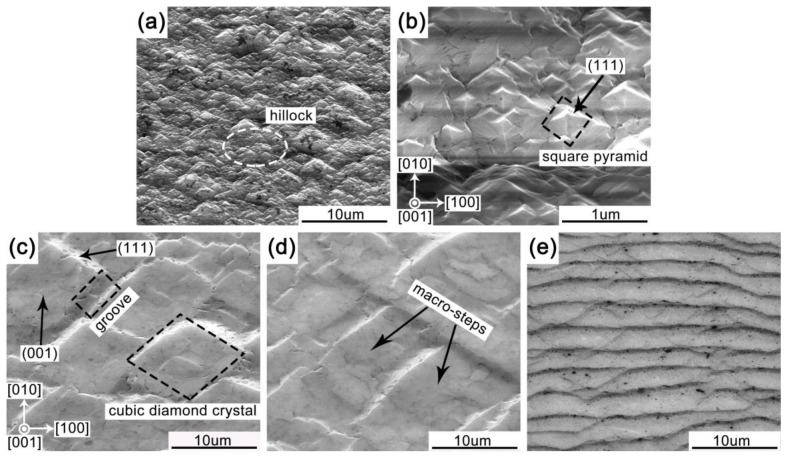
(**a**) SEM image of surface morphology of hillock interlayer; (**b**) magnified SEM image from the area marked with ellipse in (**a**); (**c**–**e**) SEM images of diamond surface after step-flow growth for 10 min, 20 min, and 40 h, respectively.

The hillock interlayer with thickness of 90 μm was deposited on the surface of HPHT substrate under hillock growth parameters. Figure 1a presents the SEM image of HPHT substrate with grown hillock interlayer. It can be noticed that a coarse surface morphology with isolated particles was obtained, and isolated particles gather together forming small hillock features. The formation of hillocks is attributed to repeated two-dimensional nucleation on diamonds. As shown in Figure 1b, the SEM image shows that the surface of hillock interlayer consists of square pyramids with (111) faces, and the base length of medium sized square pyramidal crystallite was approximately 250 nm.

To explore how a step-flow SCD film was deposited on hillock interlayer under step-flow growth parameters, the surface morphology evolution as a function of deposition time was investigated. Figure 1c–e exhibits the SEM images of sample surface morphology evolution with deposition time extension of step-flow growth. As shown in Figure 1c, after the step-flow SCD deposited on hillock interlayer for 10 min, the cubic diamond crystals were observed. The cubic diamond crystals were composed of (001) and (111) faces, and the smooth square portions were (001) faces. As deposition time ran, the pyramidal hillocks significantly grew and resulted in the merging of adjacent pyramids and the generation of steps, and the (001) faces were formed on the top of the square pyramidal crystals (Figure 1b) by lateral growth in the [110] and [1–10] directions, leading to a change of surface morphologies from hillocks to macro-steps. When the deposition time of step-flow growth was extended to 20 min, as shown in Figure 1d, the macro-steps appeared while the isolated cubic diamond crystals disappeared. The grooves formed by the coalescence of (001) faces disappeared on the diamond growth surface. That means the lateral growth of (001) was faster than that of other faces. It could be considered that the lateral movement of reaction radicals through the continuous supply of hydrocarbon precursors into terrace steps makes a major contribution to the growth of (001) homoepitaxial diamond. Then, the cubic diamond crystals with (001) faces are bunched together to cause macro-steps. With deposition time of step-flow growth extension, the hydrocarbon adsorbates arriving at terraces diffused at the edges and then incorporated into the lattice, resulting in the step-flow growth (Figure 1e). When the deposition time was extended to 40 h, a continuous SCD growth surface appeared and high-density uniform step-bunching could be clearly observed, which indicates a typical step-flow growth model with N_2_ introduction [32], as shown in Figure 1e.

**Figure 2:**
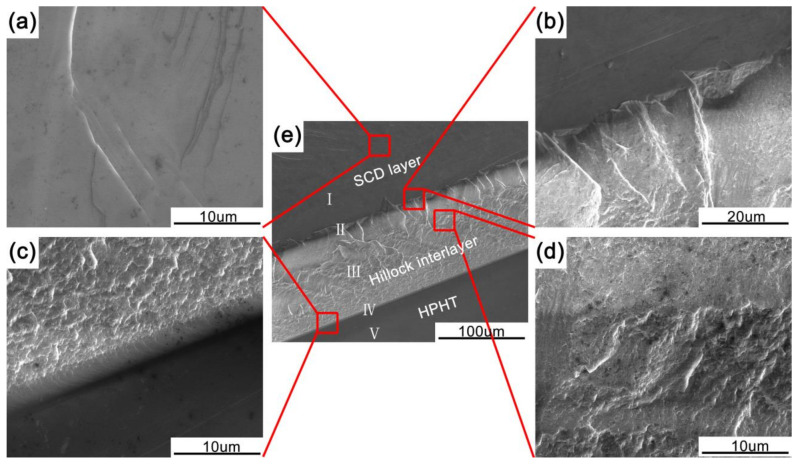
Cross-section SEM images of SCD layer/hillock interlayer/HPHT diamond structure. (**a**) Magnified SEM image of SCD layer; (**b**) magnified SEM image of the interface between the hillock interlayer and SCD layer; (**c**) magnified SEM image of transition region between the HPHT and hillock interlayer after the hillock growth; (**d**) magnified SEM image of hillock interlayer; (**e**) and cross-section SEM image of the homoepitaxial grown diamond, wherein the location with Roman numeral marker indicated the measure position of Raman and PL.

In order to trace the structural transformation, the cross-section SEM images of morphological and structural evolution were characterized. Figure 2e shows the SEM image of cross-section structure, wherein the positions with Roman numeral marks indicated the measurement locations of Raman and PL spectra. The cross-section topography obviously exhibits different crystalline structure. The changes in morphology are associated with the difference of growth parameters. Three different layers have been categorized: HPHT substrate, hillock interlayer, and SCD layer. Figure 2a exhibits a uniform and continuous layer. Considering the results shown in Figure 1e, it could be thought that this layer is an SCD [33]. Figure 2b shows an interface between the hillock interlayer and SCD layer. The upside dark region indicates the SCD layer, and the downside grey region shows the hillock interlayer. It could be clearly observed that when the growth model was changed to step-flow growth parameters, the compactness of hillock interlayer was gradually improved, resulting in a transformation of diamond layers from hillock interlayer to SCD layer. As shown in Figure 2d, a granular morphology has been observed in hillock interlayer. Figure 2c illustrates that there existed a dense and uniform transition region between the HPHT and hillock interlayer after the hillock growth; this is because the diamond substrate surface is flat and well-polished, on which the hillocks were formed and indicated a dense and uniform morphology. When the growth time was longer, the hillocks became larger and subsequent layer grew on the coarse three-dimensional surface of the previous one.

**Figure 3:**
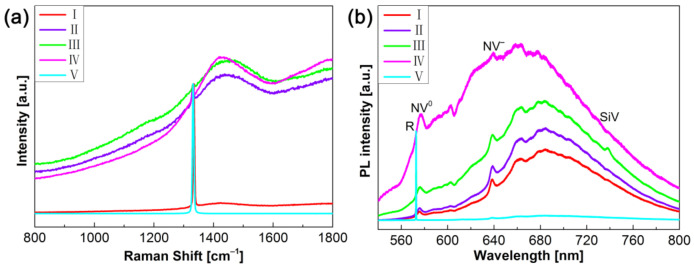
(**a**) Raman and (**b**) PL spectra of cross-section SCD layer/hillock interlayer/HPHT diamond structure. The Roman numerals of I, II, III, IV and V correspond to the positions for taking the Raman and PL spectra as indicated in Figure 2e.

Raman spectroscopy is a powerful technique to assess the crystal quality of epitaxial layers and identify sp^3^ bonding in diamonds from sp^2^ in graphite and other types of carbon allotropes [34], and Raman spectroscopy is very sensitive to the presence of non-diamond carbon, which may be codeposited simultaneously during diamond growth. As shown in Figure 3, Raman and PL measurements were carried out at several points marked as I, II, III, IV and V in Figure 2e. Raman results in Figure 3a indicate that the characteristic Raman peak of diamond appears in all spectra, where spectra are normalized by their own diamond peak intensity, respectively. It is to be noticed that there is a broad peak near the diamond peak around 1420 cm^−1^ (points I, II, III, and IV), which could be attributed to the fluorescence owing to the nitrogen-vacancy center [32,35,36,37,38]. The intensity of fluorescence of the nitrogen-vacancy center tends to decrease when the growth conditions are changed to step-flow growth parameters. Moreover, the step-flow SCD layer (point I) presents a strong and sharp diamond peak at 1334.39 cm^−1^ with the full width at half maximum of 4.82 cm^−1^, while it is 1331.19 cm^−1^ with the full width at half maximum of 5.92 cm^−1^ of the HPHT substrate (point V). It could be thought that despite the repetitive growth, the crystal quality of step-flow SCD layer was not degraded by introducing a hillock interlayer.

To further investigate the types of impurities and defects in the synthesized diamond, PL spectral evolution was employed in the cross-section along growth direction. As illustrated in Figure 3b, the intensity of the PL spectra was normalized to the diamond Raman peak R at 572 nm, aiming at exploring changes in color centers with different growth models. Besides the Raman peak R, the typical zero phonon lines are observed at 575 nm and 638 nm (point I, II, III, IV), which can be attributed to the neutral (NV^0^) and negatively charged states (NV^−^) of nitrogen-vacancy center, respectively [32,39,40,41,42]. It should be noted that the PL peak related to silicon-vacancy (SiV) center could be found at 738 nm (point III) [32,39]. Furthermore, the intense broad band is presented in the region of 600–800 nm (point I, II, III, IV), which is assigned to the associated phonon replicas of nitrogen-vacancy related zero phonon lines [32,39,40,41,42]. Obviously, the intensity of the broad band recorded in the center region is monotonically decreased in the cross-section in a growth direction. In addition, the PL spectra of HPHT substrate (point V) was dominated by the diamond Raman peak R, and a weak NV^−^ center could be observed. Considering the Raman and PL results, the incorporation probability of N_2_ in the diamond depends on the growth sector, and the N_2_ was preferentially incorporated in the {111} growth sectors in hillock interlayer [43,44].

A detailed study of the grown diamond microstructure under step-flow growth parameters may obtain information about the layer components and structures, which could help us to gain deeper insight into the growth mechanism of epitaxial diamond films. For this purpose, the high-magnification HRTEM images and selected area electron diffraction patterns were performed at a random area in the grown diamond under step-growth parameters.

**Figure 4:**
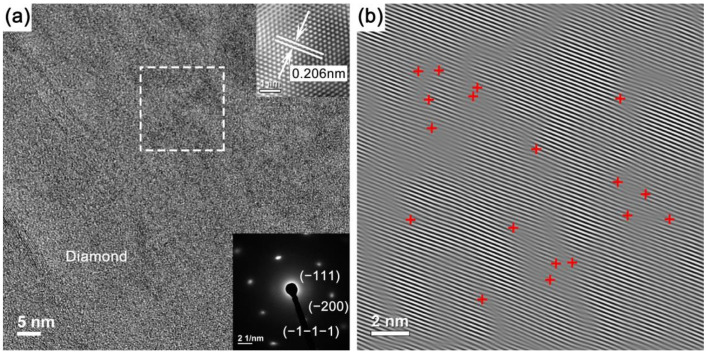
(**a**) High-magnification cross-section HRTEM image of SCD layer near the hillock interlayer with selected area filtered HRTEM micrograph (top right insert), and the bottom right insert shows a typical selected area electron diffraction pattern; (**b**) inverse fast Fourier transform image of selected area marked by white-dashed box in (**a**), and the red cross indicates the dislocations.

Figure 4a shows the high-magnification cross-section HRTEM image of SCD layer near the hillock layer, and the interplanar spacing obtained from selected area filtered HRTEM micrograph (top right insert of Figure 4a) is 0.206 nm, corresponding to the diamond (111) plane. As shown in the bottom right insert of Figure 4a, a face-centered cubic crystal lattice along the [0–11] zone axis could be observed. The interplanar spacing of the selected area electron diffraction spots was measured and matched the diamond crystal plane characteristics. The deduced interplanar spacing from the strong diffraction spots corresponding to {220}, {111}, and {200} crystal planes was 0.128 nm, 0.219 nm, and 0.175 nm, respectively. Referring to the parameters of high-quality SCD in the X-ray diffraction database (PDF#06-0675), the corresponding interplanar spacing was 1.261 Å, 2.060 Å, and 1.783 Å, respectively, indicating that experiment results are almost identical to the database.

**Figure 5:**
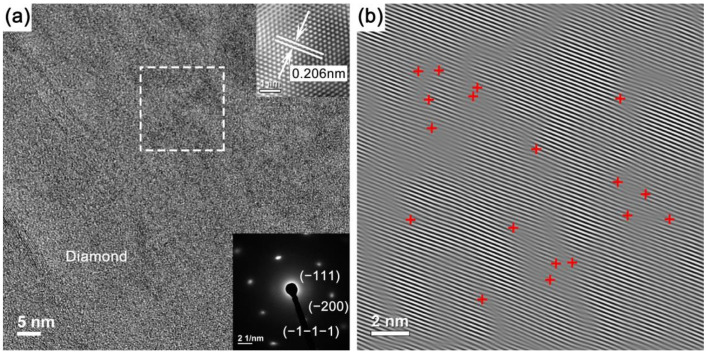
(**a**) High-magnification HRTEM image of surface SCD layer with selected area filtered HRTEM micrograph (top right insert), and the bottom right insert shows a typical selected area electron diffraction pattern; (**b**) inverse fast Fourier transform image of selected area marked by white-dashed box in (**a**).

The high-magnification HRTEM image and selected area electron diffraction pattern of surface SCD layer are shown in Figure 5a. The interplanar spacing obtained from selected area filtered HRTEM micrograph (top right insert of Figure 5a) is 0.128 nm, corresponding to the diamond (220) plane. The selected area electron diffraction pattern (bottom right insert of Figure 5a) demonstrates the single crystal structure with a face-centered cubic crystalline lattice of the diamond, and the zone axis is along the (001) direction. The diffraction spots are indexed to the {220} and {400} crystal planes with interplanar spacing of 0.136 nm and 0.096 nm, respectively. Referring to the parameters of high-quality SCD in the X-ray diffraction database (PDF#06-0675), the corresponding lattice spacing was 1.2610 Å and 0.8916 Å, respectively.

It was found that the selected area electron diffraction spots in Figure 4a were more oval and larger than those of Figure 5a, indicating some point defects and a slightly disordered region in the SCD layer near the hillock interlayer. Figure 4b and Figure 5b are the corresponding inverse fast Fourier transform images. Compared with Figure 4b, the lattice arrangement in Figure 5b exhibits much better order, which means that with deposition time of step-flow growth extension, the crystal structure of the SCD layer became quiet complete without evident indications of dislocation structure.

## 4. Conclusions

In summary, homoepitaxial growth of step-flow SCD layer on hillock interlayer was performed by a microwave plasma chemical vapor deposition system. Under a hillock growth model, a coarse surface morphology with isolated particles was observed, and isolated particles gathered together forming small hillock features. When the growth conditions were changed to step-flow growth parameters, the surface morphology varied from growth hillocks to macro-steps, and then a continuous step-flow SCD layer with high-density uniform step-bunching was obtained. Despite the repetitive growth, the crystal quality of step-flow SCD layer was not degraded by introducing a hillock interlayer. Furthermore, the as-grown diamond under step-flow growth parameters matched the single crystal structure with a face-centered cubic crystal lattice of the diamond. It was also found that there were some point defects and a slightly disordered region in the SCD layer near the hillock interlayer. With deposition time extension, the crystal lattice of the SCD layer became complete.

## Data Availability

Data is contained within the article.
